# Phyllosphere microbial diversity and specific taxa mediate within-cultivar resistance to *Phytophthora palmivora* in cacao

**DOI:** 10.1128/msphere.00013-23

**Published:** 2023-08-21

**Authors:** Jennifer E. Schmidt, Alina S. Puig, Ashley E. DuVal, Emily E. Pfeufer

**Affiliations:** 1 Cocoa Plant Sciences, Mars Wrigley, Davis, California, USA; 2 Foreign Disease-Weed Science Research Unit, USDA-ARS, Fort Detrick, Frederick, Maryland, USA; E O Lawrence Berkeley National Laboratory, Berkeley, California, USA

**Keywords:** biocontrol, black pod disease, phyllosphere microbiome, *Phytophthora palmivora*, plant-microbe interactions, *Theobroma cacao*, oomycete pathogens

## Abstract

The oomycete pathogen *Phytophthora palmivora*, which causes black pod rot (BPR) on cacao (*Theobroma cacao* L.), is responsible for devastating yield losses worldwide. Genetic variation in resistance to *Phytophthora* spp. is well documented among cacao cultivars, but variation has also been observed in the incidence of BPR even among trees of the same cultivar. In light of evidence that the naturally occurring phyllosphere microbiome can influence foliar disease resistance in other host-pathogen systems, it was hypothesized that differences in the phyllosphere microbiome between two field accessions of the cultivar Gainesville II 164 could be responsible for their contrasting resistance to *P. palmivora*. Bacterial alpha diversity was higher but fungal alpha diversity was lower in the more resistant accession MITC-331, and the accessions harbored phyllosphere microbiomes with distinct community compositions. Six bacterial and 82 fungal amplicon sequence variants (ASVs) differed in relative abundance between MITC-333 and MITC-331, including bacterial putative biocontrol agents and a high proportion of fungal pathogens, and nine fungal ASVs were correlated with increased lesion development. The roles of contrasting light availability and host mineral nutrition, particularly potassium, are also discussed. Results of this preliminary study can be used to guide research into microbiome-informed integrated pest management strategies effective against *Phytophthora* spp. in cacao.

**IMPORTANCE:**

Up to 40% of the world’s cacao is lost each year to diseases, the most devastating of which is black pod rot, caused by *Phytophthora palmivora*. Though disease resistance is often attributed to cacao genotypes (i.e., disease-resistant rootstocks), this study highlights the role of the microbiome in contributing to differences in resistance even among accessions of the same cacao cultivar. Future studies of plant-pathogen interactions may need to account for variation in the host microbiome, and optimizing the cacao phyllosphere microbiome could be a promising new direction for *P. palmivora* resistance research.

## INTRODUCTION

Chocolate is made from the seeds of *Theobroma cacao*, a tree grown commercially in tropical regions around the world. Up to 40% of the $12 billion global harvest is lost to diseases (1), the most significant of which is black pod rot (BPR), caused by species of *Phytophthora*. Numerous *Phytophthora* species have been found to cause BPR (*P. megakarya*, *P. palmivora*, *P. capsici*/*P. tropicalis*, and *P. theobromicola*), with infection typically occurring through motile zoospores released in the presence of free water. Infected pods become completely necrotic within weeks (2).

Scientists and breeders are working to increase the supply of cacao beans by improving the management of BPR in the field and developing disease-resistant cultivars. Cacao germplasm and newly developed varieties are evaluated to determine disease resistance using field trials in various locations worldwide and through direct inoculation of fruits (3 – 5). Although numerous *Phytophthora* species have been found to cause BPR, only *P. palmivora* is present in all commercial production areas, and thus, it is most frequently used when screening for disease-resistant germplasm (5, 6).

Conflicting levels of host resistance have been obtained by different scientists for specific cacao cultivars. These discrepancies could be attributed to genotype × environment interactions, including differences in mineral nutrition, or to germplasm mislabeling. Variation in nutrient concentrations of tissues has shown to be correlated with susceptibility or resistance of various plant species to pathogens (7). In cacao, this response has also been inferred from the decrease in severity of fungal and viral symptoms upon application of foliar fertilizers (8). A study of cacao seedling response to *P. megakarya* showed negative correlations between disease severity and sulfur-containing compounds cysteine and glutathione, highlighting the importance of controlling for mineral nutrition as a potential confounding factor in pathogenicity screens (9). However, substantial variation in resistance is also observed among pods from the same tree (A. Puig, unpublished data), suggesting that factors beyond mineral nutrition may play a role.

Differences in *Phytophthora* resistance between cacao trees could also be caused by differences in the composition of phyllosphere and endophytic microbial communities. Plant-associated microorganisms have been associated with improved pathogen resistance or reduced disease severity in numerous host-pathogen systems (10). Endophytic microorganisms are particularly important given their residence within the host plant, where they can produce metabolites that influence plant signaling pathways, chemical composition, and physiology (11, 12). Plant endophytes have also been suggested as potential biocontrol agents specifically for *Phytophthora* spp., through mechanisms including predation, parasitism, niche competition, or chemical inhibition (13).

Beneficial effects of plant microbiomes on disease resistance can generally be divided into two categories: biocontrol agents, in which individual microbial strains inhibit a pathogen, and disease-suppressive microbiomes, in which features of the microbiome as a whole protect the plant against disease (14, 15). Cacao-specific biocontrol research has addressed many of its most damaging pathogens: *Phytophthora* spp. (16 – 27), *Moniliophthora* spp. (28 – 32), and *Colletotrichum* spp. (33). Despite some success, application of exogenous biocontrol agents may only provide short-term disease suppression [e.g., 50% protection at 5 weeks, with no measurable effect after 7 weeks (17)].

The alternative strategy of cultivating disease-suppressive microbiomes seeks to deliver longer-lasting effects by optimizing overall diversity or other community-level properties, but studies are more limited in cacao. Arnold et al. showed that inoculating endophyte-free leaves with a diverse leaf endophyte microbiome naturally occurring in disease-free cacao trees provided protection against pathogen challenge (34). Similarly, Christian et al. showed that transferring leaf litter (with the associated phyllosphere microbiome) from healthy cacao trees led to improved disease resistance in seedlings (35). Still, knowledge gaps remain as to which specific phyllosphere-inhabiting microbial taxa or microbiome features might provide protection against *Phytophthora* spp. in cacao.

This preliminary study compared two field-grown accessions of a single cacao clone that contrasted in resistance to *P. palmivora* to identify potentially protective taxa or phyllosphere microbiome properties. The accessions were hypothesized to harbor microbial communities that differed in the relative abundance of key protective bacteria or fungi and/or in microbiome features such as diversity or aspects of community composition that could improve *Phytophthora* resistance.

## MATERIALS AND METHODS

### Plant material

Immature, open pollinated cacao pods (4–5 months old) were collected from two different field accessions (MITC-331 and MITC-333) of the cultivar Gainesville II 164 (GNV-164 II) growing in close proximity (<7 m apart) at the germplasm collection at the USDA-ARS Subtropical Horticulture Research Station in Miami, Florida. Both trees were 20 years old and had been grafted onto open pollinated rootstock from the cultivar EET-400. Despite being in close proximity, MITC-331 receives full sun, while MITC-333 receives partial sun. MITC-331 produces dark red pods, as is typical of this genotype in full sun conditions, and MITC-333 produces pods that are mostly green-pigmented (36).

### Pod disease severity screening

To determine the resistance of pods from each field accession, unwounded pod husk inoculation assays were carried out with zoospores of a *P. palmivora* isolate, H33, collected from Hawaii (37). Zoospore inoculum was produced using a protocol modified from Ali et al. (38). Five-millimeter diameter plugs from the margins of 5-day-old *P. palmivora* colonies growing on 20% V8 agar were placed mycelial side down in the center of new 20% V8 agar plates, then maintained for 5 days in darkness followed by 5 days under continuous light in a 25°C incubator. Zoospore release was induced by adding 12 mL cold sterile water (4°C), then placing plates at 4°C for 45 minutes, followed by 30 minutes at 28°C. Zoospore concentrations were calculated using disposable KOVA Glasstic Slide 10 (KOVA International Inc., Garden Grove, CA, USA).

Each pod was inoculated at a single site with 2,000 zoospores (in a 50 µL solution), which was secured by placing a 1 × 1 cm^2^ filter paper disc on the inoculum. A subset of pods inoculated with sterile water served as the negative controls. Each pod was placed in an individual sealed plastic bag and incubated at 25°C. Two lesion radii were measured per pod at 5, 6, and 7 days post inoculation, and used to calculate lesion area at each time point (39). To confirm that the resulting lesions were caused by *P. palmivora*, isolations were made from a subset of lesions as described in Puig et al. (37). The assay was carried out as a completely randomized design, with MITC-331 (full sun) and MITC-333 (partial sun) represented by 13 and 5 pods (replicates), respectively, determined by availability.

Area under the disease progress curve (AUDPC) was calculated using IdeTo, an Excel-based calculator (40), and analyzed in SAS 9.4 using a non-parametric t-test to determine differences between pods from different trees.

### Leaf inoculations and tissue sampling

Expanded, tender leaves from MITC-333 and MITC-331 were divided in half, lengthwise along the midvein, with one half being further subdivided into 90 mm Petri dishes containing damp Whatman No. 2 filter paper (two to three subsections depending on leaf size). Leaf subsections were placed abaxial side up and inoculated with mycelial plugs of *P. palmivora* growing on 20% V8 agar media, placed on the laminar tissue between minor veins. The other leaf half was placed at −80°C, lyophilized, and vacuum sealed before processing for microbial community analysis. A subset of leaf sections was inoculated with uncolonized plugs of 20% V8 agar media and served as negative controls. Plates were sealed and placed at 25°C in the dark.

Lesion length and width were measured 4 and 5 days post inoculation, and used to calculate average lesion diameter on each day. Nine to 11 leaves were used for each of the field accessions, and lesions from each subsection were averaged to obtain a single value per leaf per day. The assay was repeated on a second date and data were pooled for analysis. AUDPC was calculated and analyzed as described above. Twenty total replications (leaves) were done per field accession.

### Leaf nutrient analysis

For each of the two field accessions in the study, leaves were sampled for macro- and micronutrient analysis to account for any potential effects of plant nutrition. The third leaf from the most recently matured flush was sampled uniformly to cover all sides of the tree from the upper canopy (*n* = 5) and lower canopy (*n* = 5). Samples were pooled by canopy position and accession and submitted to Waypoint Analytical (Richmond, VA, USA) for leaf tissue nutrient analysis of 13 macro- and micronutrients according to the methods of Bryson et al. (41).

### Molecular methods

Phyllosphere microbiome DNA [including both phylloplane and endophytic microorganisms (42)] was extracted from six leaf punches per sample using a DNeasy PlantPro kit (QIAGEN, Germantown, MD, USA). The lysis step was performed on a PreCellys Evolution (Bertin Instruments, Montigny-le-Bretonneux, France) with 3 × 15 s cycles at 5,000 rpm. DNA was stored at −80°C until library preparation and sequencing. PCR amplification of the 16S V4 region was performed by using the original 515F/806R primer pair (43) ligated with barcodes. For fungal communities, the ITS1F/ITS2 primer pair was used to amplify the ITS1 region (44, 45). The PCR products of the correct length were selected by 2% agarose gel electrophoresis. An equivalent amount of PCR products from each sample was pooled, end-repaired, A-tailed, and further ligated with Illumina adapters. The library was checked with Qubit and real-time PCR for quantification and bioanalyzer for size distribution detection. Quantified libraries were pooled and sequenced on an Illumina NovaSeq platform to generate 250 bp paired-end raw reads. The sequencing data were deposited in the NCBI Sequence Read Archive database under the project ID PRJNA925518.

### Bioinformatics and microbial community analysis

Adapters were trimmed, paired-end reads were demultiplexed by matching barcodes to sample names, and barcodes were removed. Reads with a mean quality score of 21 or less were filtered out. Downstream analyses were all conducted in R v.4.1.3 (46), and are described using package::function() here. The dada2 pipeline was used for sequence denoising, quality filtering (parameters: maxN = 0, maxEE = c (2, 2), truncQ = 2), removal of 16S sequences outside the expected length of 250–256 bp, and generation of amplicon sequence variant (ASV) tables (47). The phyloseq package was used to remove chloroplast and mitochondria sequences and construct phyloseq objects for further analysis (48). Unrooted phylogenetic trees were constructed using the DECIPHER package v.2.22.0 (49) and the phangorn package v.2.8.1 (50), with the general time-reversible model and nearest neighbor interchange rearrangement.

Three metrics of alpha (within-sample) diversity were calculated using the vegan package v.2.5.7 (51). Richness was calculated as the number of unique ASVs per sample (vegan::specnumber()), and the Shannon index, representing community richness and evenness, was calculated with vegan::diversity() with index = “shannon”. The Pielou index, a measurement of evenness, was then calculated as the Shannon index divided by the logarithm of species richness. Faith’s PD, an alpha diversity metric that takes phylogenetic information into account, was calculated using the metagMisc package v.0.0.4 (metagMisc::phyloseq_phylo_div() with measures = “PD”) (52). Differences in phyllosphere microbiome alpha diversity between trees were tested with a Student’s t-test.

Canonical analysis of principal coordinates (CAP) based on Bray-Curtis distance and weighted UniFrac was used to ordinate phyllosphere communities. Microbial ASV tables were converted to relative abundance (53) and vegan::ordinate() was implemented with tree ID and sampling date as fixed effects. Permutational analysis of variance (PERMANOVA) with 5,000 permutations was used to test fixed effects with vegan:adonis2().

The package MaAsLin2 (54), which allows the construction of microbiome-specific generalized linear models, was used to screen for associations with parameters of interest. Separate univariate models were constructed for field accession, lesion size at day 5, and AUDPC. Stringent analysis parameters were used: the minimum prevalence threshold was set at 25% and maximum q value at 0.01 (i.e., controlling the Bonferroni-Holm-adjusted false discovery rate at 0.01) and results were filtered afterward to keep only taxa with *P* < 0.01 (i.e., controlling the rate of false positives at 0.01). The microeco package (55) was used to classify fungi that differed in abundance between trees according to functional guilds using the FUNguild database (56) and FungalTraits database (57). FUNGuild assigns fungal sequences to guilds based on taxonomic affinity at the genus or species level, and further specifies a level of confidence (“possible,” “probable,” or “highly probable”) for each assignment (55). The FungalTraits database, released 5 years later, builds on FUNGuild and other previously published fungal trait datasets and includes both primary and secondary lifestyle classifications, but also relies on taxonomic assignment at the genus and species levels. These classifications can be found in the supplementary materials.

## RESULTS

### Lesion development on pods and leaves

**Fig 1:**
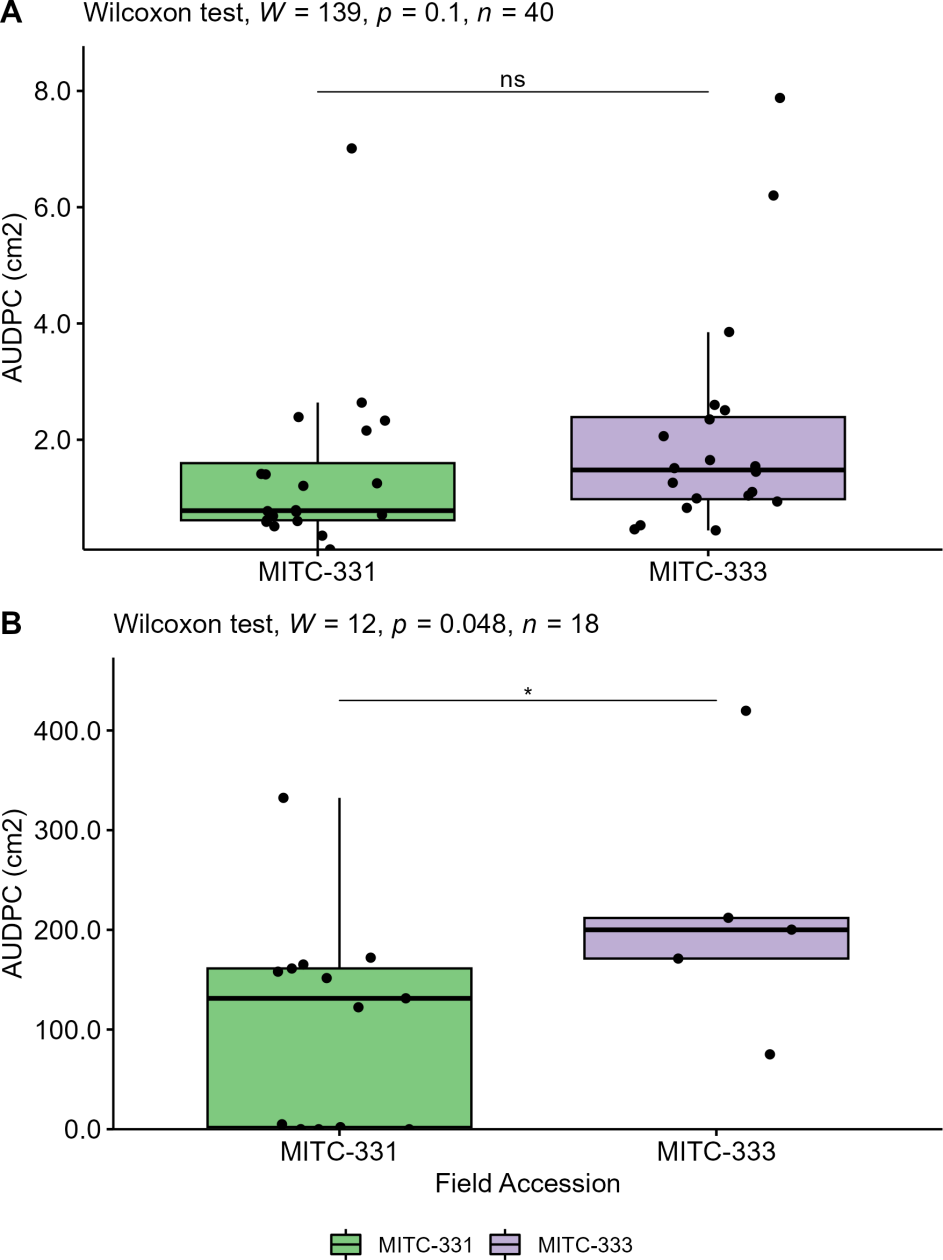
Lesion development on leaves and pods of two cacao field accessions. (A) On leaves inoculated with *P. palmivora,* the AUDPC did not differ significantly between two accessions (MITC-331 and MITC-333) of the cultivar Gainesville II 164 at the α = 0.05 level. (B) The AUDPC was greater on pods sampled from MITC-333 than pods sampled from MITC-331 (Wilcoxon *P* < 0.05).

**Fig 2:**
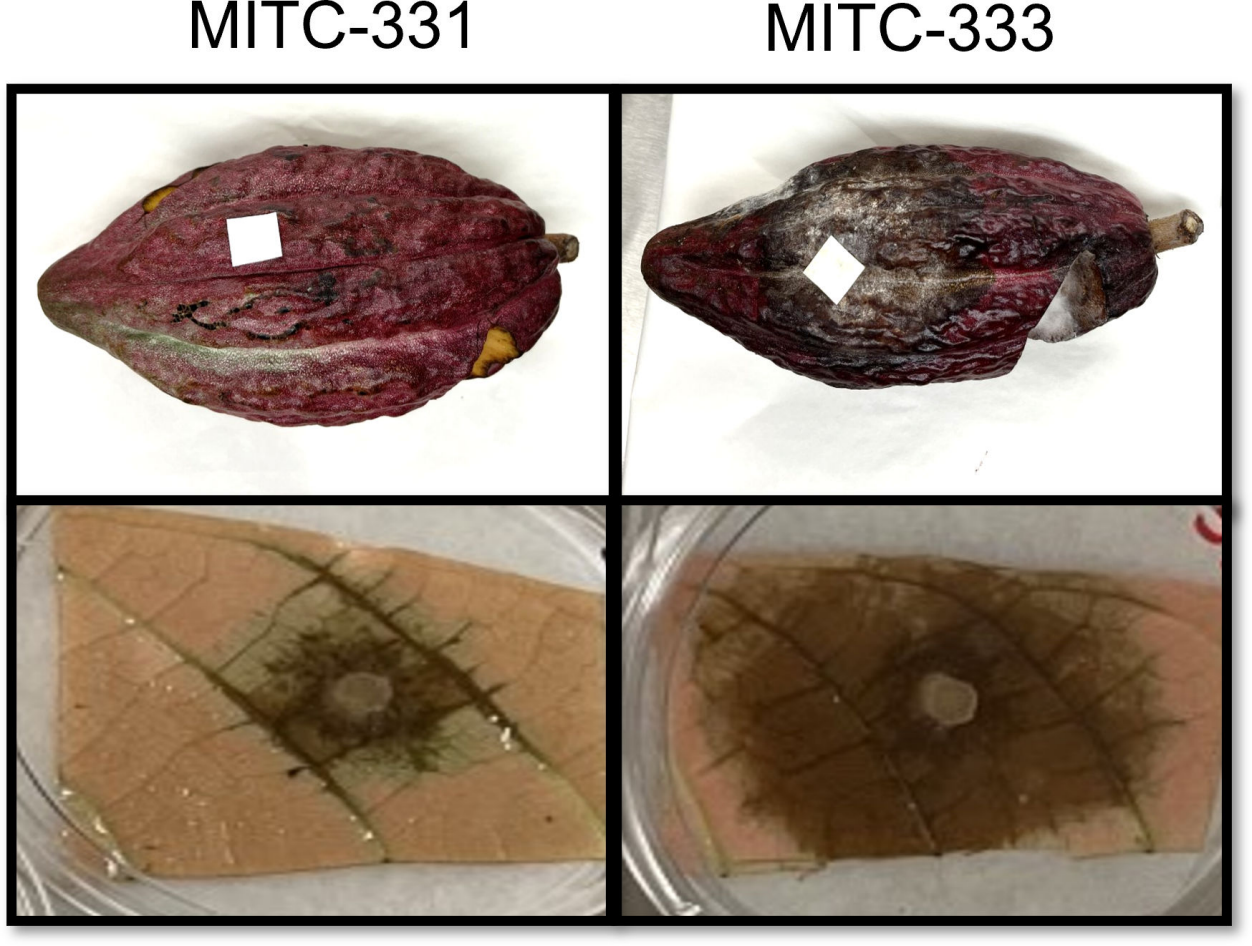
Representative photographs of inoculated pods and leaves from MITC-331 and MITC-333, two accessions of Gainesville II 164. Pods were inoculated with 2,000 zoospores of *P. palmivora*, and leaves were inoculated with discs of V8 agar media containing *P. palmivora* mycelia. A proportion of pods from MITC-331 did not develop necrotic lesions post-inoculation.

Lesion development (AUDPC) on pods and leaves following inoculation with *P. palmivora* was compared using the Wilcoxon rank sum test. Although lesions on leaves of MITC-333 tended to be larger than those on MITC-331 (Fig. 1A), with mean AUDPCs of 2.1 cm^2^ (±0.43) and 1.4 cm^2^ (±0.34), respectively, these differences were not statistically different (*P* = 0.10). However, significant differences were found between pods of MITC-331 and MITC-333 (*P* = 0.048), with larger lesions developing on the latter (Fig. 2). Mean AUDPC for MITC-331 was 107.8 cm^2^ (±28.0), compared to 215.6 cm^2^ (±56.4) for MITC-333 (Fig. 1B). At 5 days after inoculation, all pods from MITC-333 had necrotic lesions (*n* = 5), compared with only half of pods from MITC-331 (6 out of 13). By 7 days after inoculation, 23% (3 out of 13) of MITC-331 pods still had not developed lesions.

### Mineral nutrition

Leaf nutrient concentrations were largely similar between MITC-331 and MITC-333 for most plant macronutrients including nitrogen, phosphorus, sulfur, calcium, and magnesium (Table 1). However, leaf potassium was 48% higher in MITC-333 at 1.35% (averaged across canopy positions) as compared to 0.91%. Leaf concentrations of many micronutrients differed between accessions, with 27% higher boron, 154% higher manganese, and 56% higher iron in MITC-333. MITC-331 had 63% higher leaf zinc concentrations.

**TABLE 1:** Leaf nutrient content of field accessions MITC-331 and MITC-333 of the cultivar Gainesville II 164

Accession canopy	MITC-333 upper	MITC-333 lower	MITC-331 upper	MITC-331 lower
Nitrogen (%)	2.65	2.33	2.62	2.4
Sulfur (%)	0.15	0.14	0.16	0.17
Phosphorus (%)	0.14	0.14	0.13	0.14
Potassium (%)	1.17	1.53	0.96	0.86
Magnesium (%)	0.64	0.54	0.5	0.57
Calcium (%)	1.29	2.28	2.09	2.35
Sodium (%)	0.02	0.05	0.03	0.04
Boron (ppm)	73	78	62	57
Zinc (ppm)	34	42	59	65
Manganese (ppm)	89	48	28	26
Iron (ppm)	97	86	65	52
Copper (ppm)	9	10	8	9
Al (ppm)	39	30	31	36

### Fungal and prokaryotic diversity metrics

Prokaryotic within-sample (α) diversity was higher in MITC-331 leaves than MITC-333 leaves for three of the four diversity metrics measured (Fig. S1A). MITC-331 leaves had higher diversity as measured by Faith’s PD (*P* = 0.00041), richness (*P* = 0.0011), and the Shannon index (*P* = 0.0093), but not the Pielou index (*P* = 0.54). In contrast, fungal alpha diversity was higher in MITC-333 leaves than MITC-331 leaves as measured by the Pielou index (*P* = 1.1e−05), richness (*P* = 0.0057), and Shannon index (*P* = 6.1e−06), but not Faith’s PD (*P* = 0.63, Fig. S1B)

### Phyllosphere community composition

**Fig 3:**
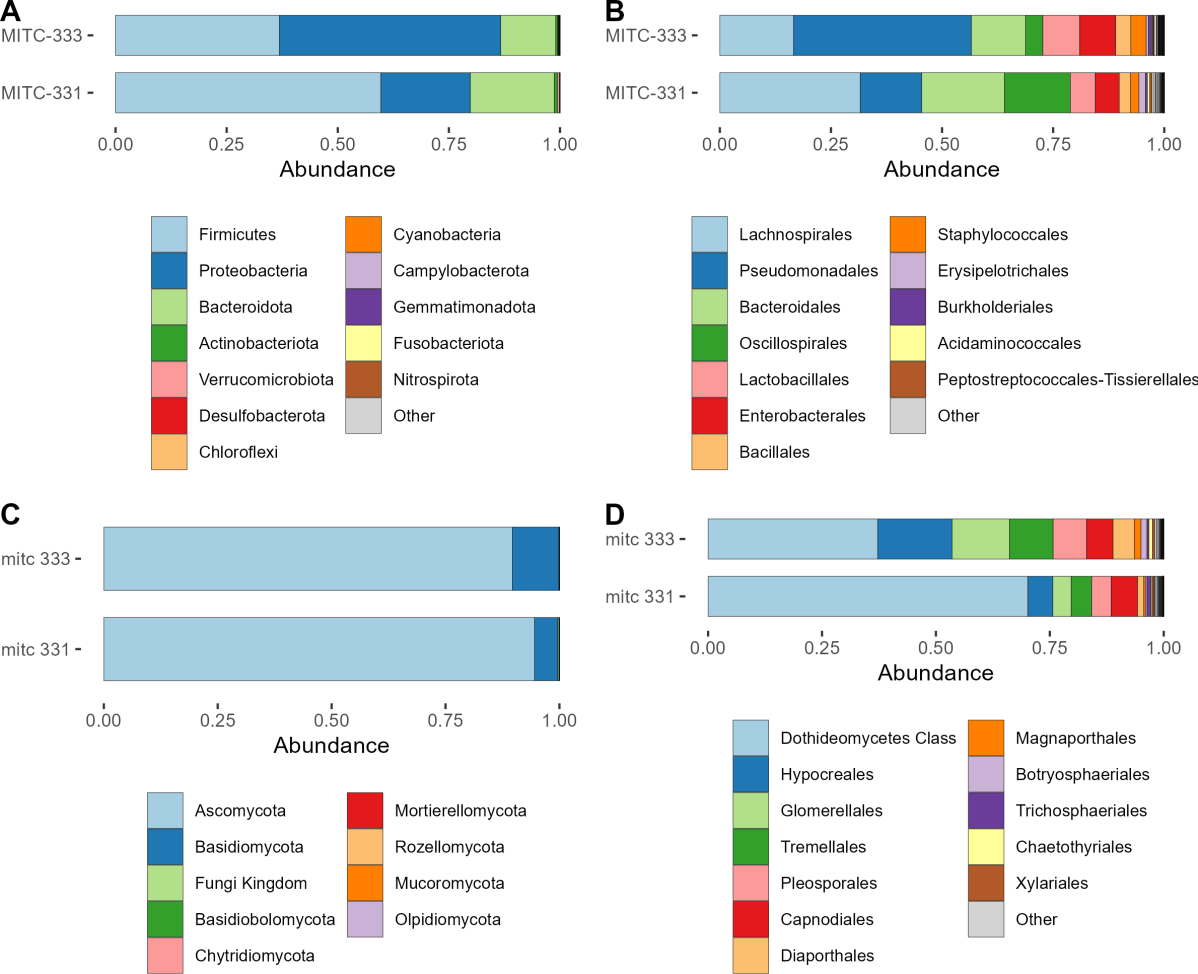
Comparison of phyllosphere microbiome composition between field accessions. (A) Prokaryotic communities at the phylum level. (B) Prokaryotic communities at the order level. (C) Fungal communities at the phylum level. (D) Fungal communities at the order level. Relative abundances are cumulative across all samples from that field accession.

Relative abundances of prokaryotic and fungal taxa differed between the two field accessions at the phylum and order levels (Fig. 3). For prokaryotic phyla, MITC-331 had higher relative abundances of Firmicutes and Bacteroidota than MITC-333, which had a higher relative abundance of Proteobacteria (Fig. 3A). At the order level, MITC-331 had higher relative abundances of Lachnospirales, Bacteroidales, and Oscillospirales and a lower relative abundance of Pseudomonodales (Fig. 3B).

Differences in fungal communities were slight at the phylum level (Fig. 3C), but more visible at the order level (Fig. 3D). The most-abundant taxon in both accessions could only be identified to the class Dothideomycetes, and its relative abundance was higher in MITC-331.

**Fig 4:**
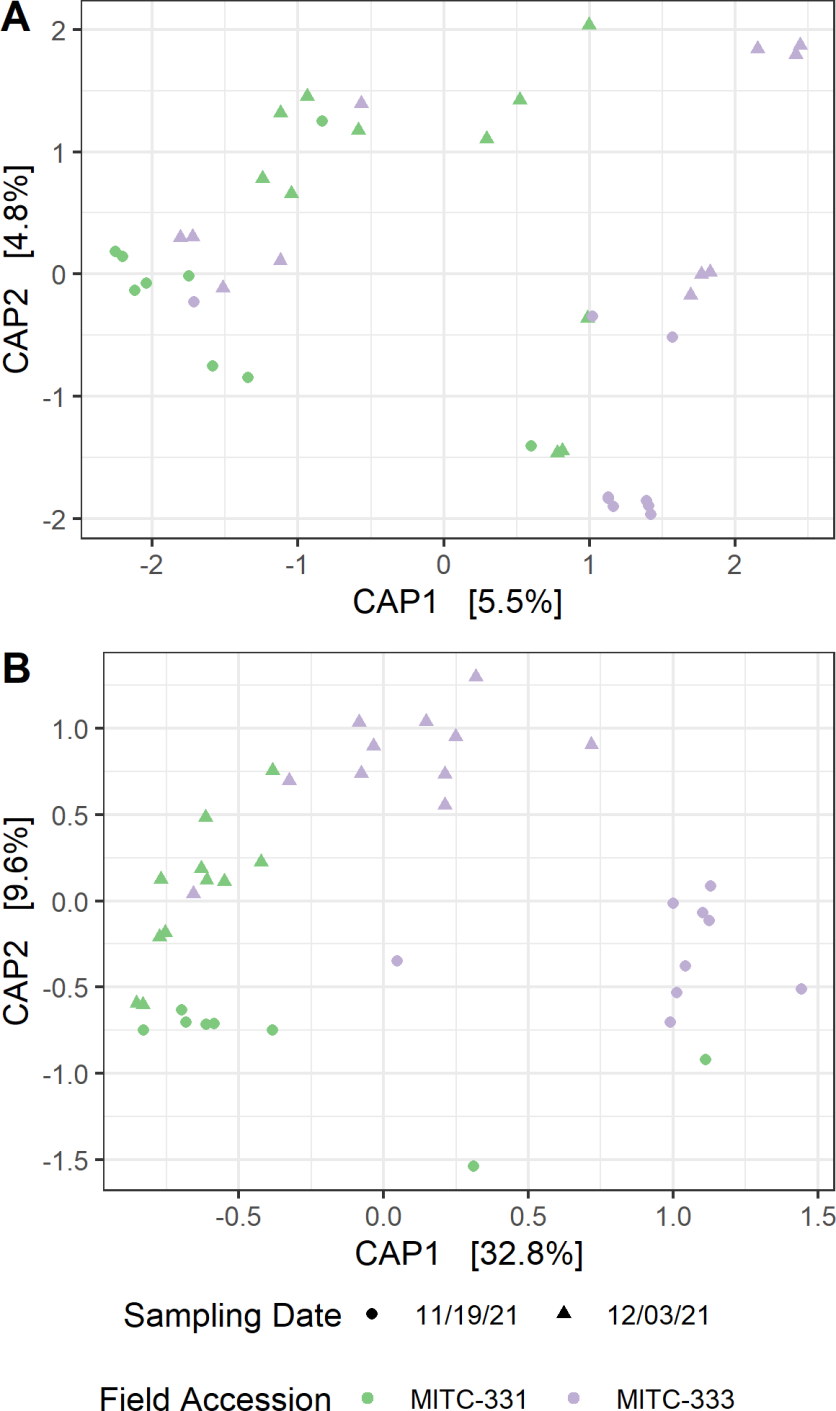
CAP of phyllosphere microbiomes. (A) Prokaryotic communities did not differ between two accessions (MITC-331 and MITC-333) of the cultivar Gainesville II 164 (PERMANOVA, *P* = 0.098) or between sampling dates (*P* = 0.11). (B) Differences were more pronounced between fungal phyllosphere communities associated with the two accessions (*P* = 0.0002) as well as between the two sampling dates (*P* = 0.0002). Ordinations were based on Bray-Curtis distance between relative abundance-transformed samples, using field accession and sampling date as fixed factors.

CAP, a constrained ordination method based on Bray-Curtis distance, was used to visualize effects of accession and sampling date on phyllosphere microbial community composition (Fig. 4). For prokaryotes, PERMANOVA showed that neither accession (*R^2^* = 0.043, *P* = 0.098) nor sampling date (*R^2^* = 0.041, *P* = 0.11) had significant effects on prokaryotic community composition (Fig. 4A). Effects of both field accession and sampling date were more pronounced for fungal communities (accession *R^2^* = 0.26, *P* = 2e−04; date *R^2^* = 0.17, *P* = 2e−04) (Fig. 4B). The ordination was repeated based on weighted UniFrac, a phylogenetic distance metric, rather than the dissimilarity-based Bray-Curtis distance, and the same patterns were observed for both prokaryotes and fungi (Fig. S2). Prokaryotic communities did not differ between accessions (*R^2^* = 0.042, *P* = 0.15) or sampling dates (*R^2^* = 0.042, *P* = 0.14), and fungal communities were affected by both accession (*R^2^* = 0.27, *P* = 2e−04) and sampling date (*R^2^* = 0.15, *P* = 4e−04).

### Microbial associations with field accessions

**Fig 5:**
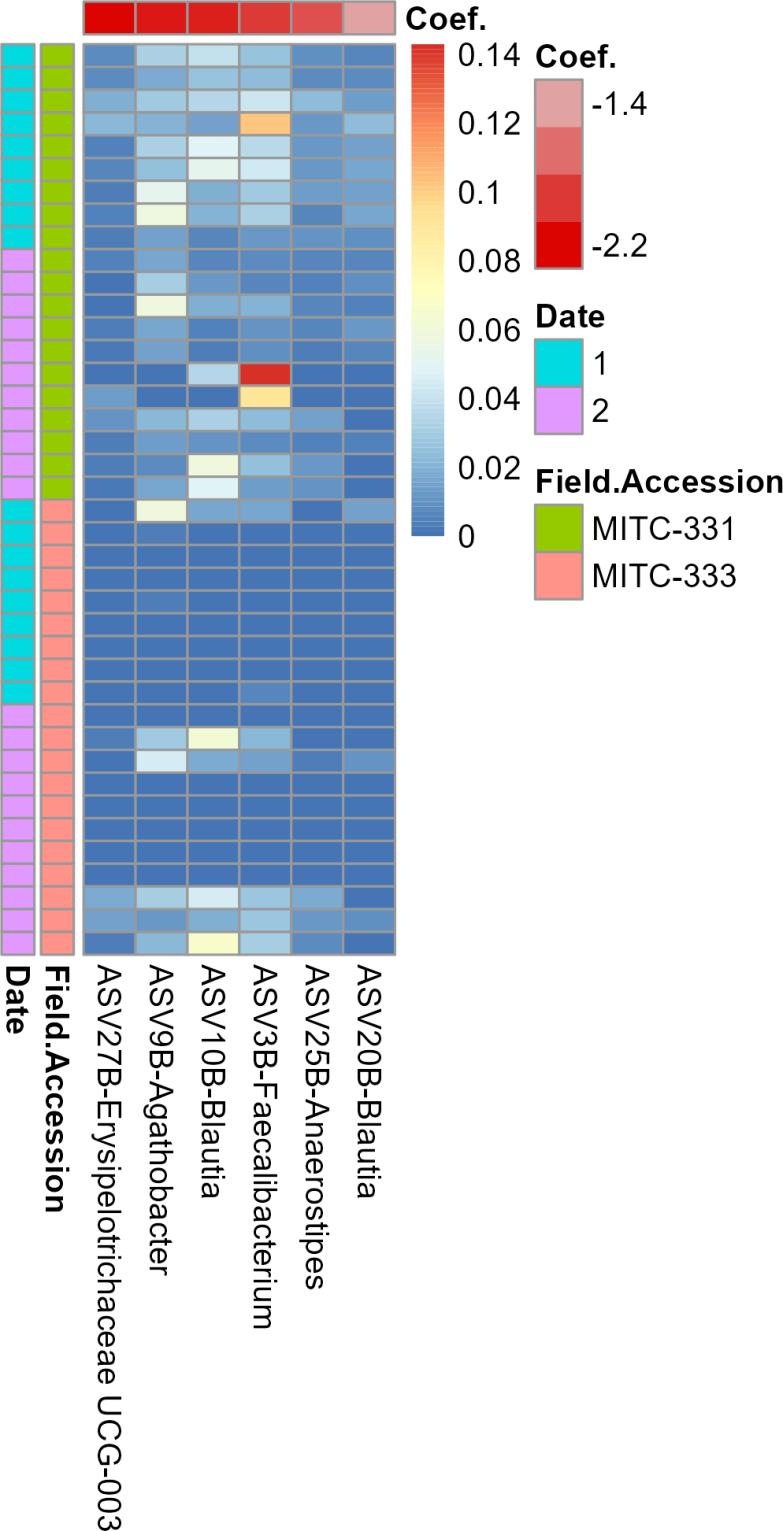
Prokaryotic taxa preferentially associated with one field accession. The heatmap shows the relative abundance of taxa (columns) across samples (rows), with red cells representing higher relative abundance and blue cells representing lower relative abundance in that sample. More negative coefficients (deeper red on top bar) indicate a higher relative abundance in MITC-331 than in MITC-333. Samples are classified to the left of the plot by field accession and sampling date. The analysis was limited to taxa with a minimum prevalence of 25% across samples, and associations were considered significant at the q < 0.01 and *P* < 0.01 thresholds.

**Fig 6:**
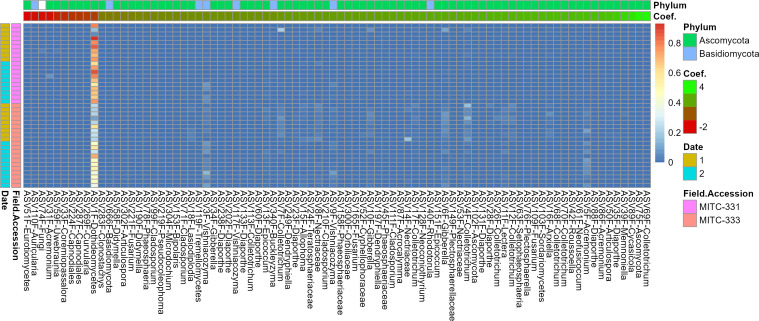
Fungal taxa preferentially associated with one field accession. The heatmap shows the relative abundance of taxa (columns) across samples (rows), with red cells representing higher relative abundance and blue cells representing lower relative abundance in that sample. Negative coefficients (red) indicate a higher relative abundance in the phyllosphere of MITC-331, while positive coefficients (green) indicate a higher relative abundance in the phyllosphere of MITC-333. Samples are classified to the left of the plot by field accession and sampling date. The analysis was limited to taxa with a minimum prevalence of 25% across samples, and associations were considered significant at the q < 0.01 and *P* < 0.01 thresholds.

Six prokaryotic and 84 fungal ASVs were associated preferentially with one of the two field accessions (Fig. 5 and 6). All six prokaryotic ASVs were found at higher relative abundance in MITC-331, with coefficients ranging from −1.24 to −2.36 (Fig. 5) (In MaAsLin2, the microbiome-specific linear regression approach used to identify differentially abundant taxa, coefficients for a categorical variable refer to the effect size). All six were classified in the phylum Firmicutes and four of the six belonged to the family Lachnospiraceae. Of the 84 fungal ASVs, 74 were more abundant in the phyllosphere of MITC-333 (Fig. 6). Seventy-four ASVs of the 84 preferentially associated with one field accession belonged to the Ascomycota and eight to the Basidiomycota. One fungal ASV belonging to the Dothideomycetes was found at far higher relative abundance than the others.

### Microbial associations with disease progression metrics

**Fig 7:**
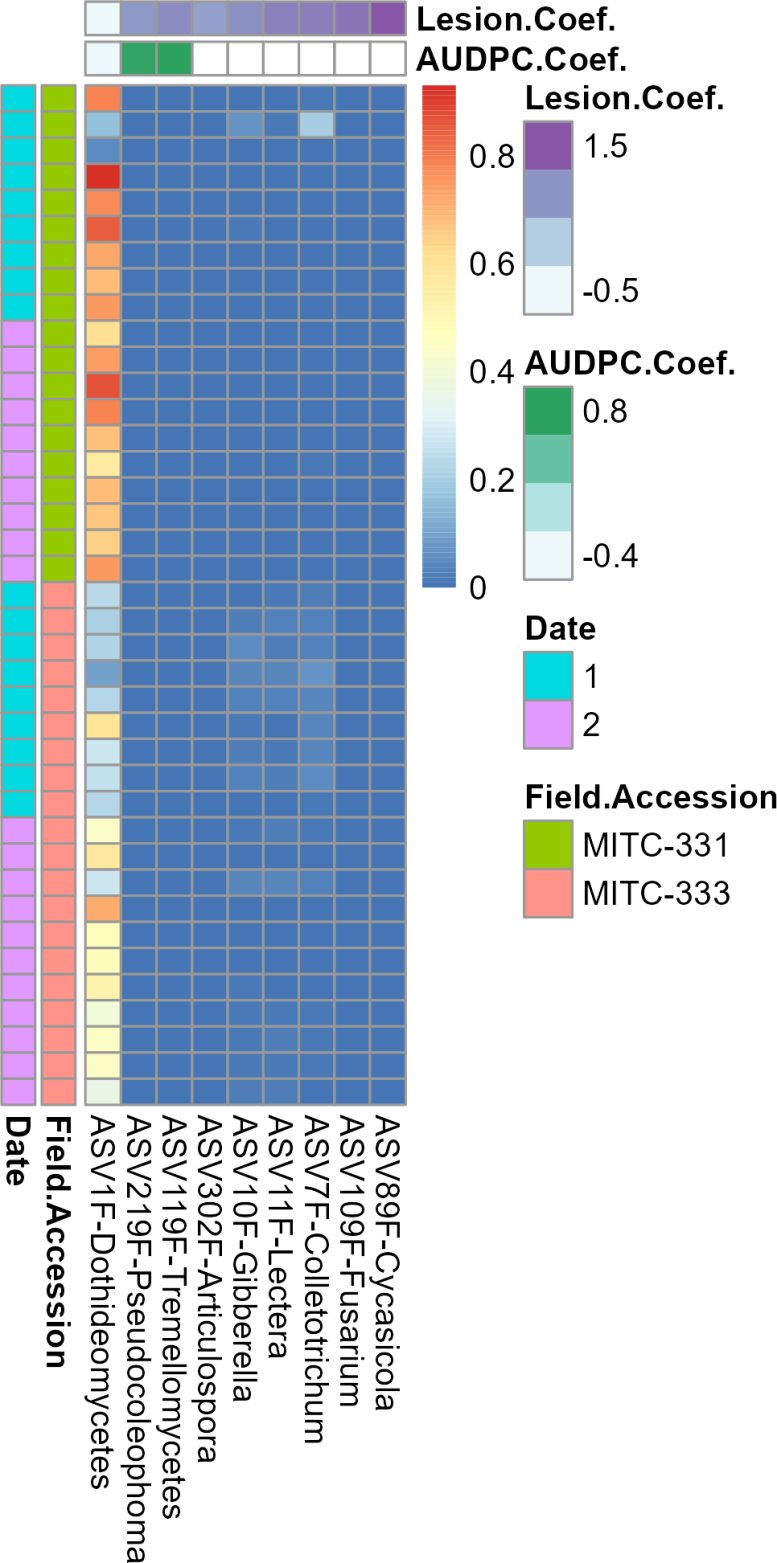
Fungal taxa associated with lesion development metrics. The heatmap shows relative abundance of taxa associated with disease (columns) across samples (rows). Red cells represent higher relative abundance and blue cells represent lower relative abundance in that sample. More positive coefficients (deeper colors on top bar) indicate a stronger positive correlation between taxon abundance and disease progression, as measured by lesion size (purple) or AUDPC (green). Samples are classified to the left of the plot by field accession and sampling date. The analysis was limited to taxa with a minimum prevalence of 25% across samples, and associations were considered significant at the q < 0.01 and *P* < 0.01 thresholds. No prokaryotic taxa were significantly associated with lesion development using these parameters.

No prokaryotic ASVs were significantly associated with either lesion size at day 5 or AUDPC. In contrast, eight fungal ASVs were associated with lesion size at day 5 and three fungal ASVs were associated with AUDPC, giving a total of 9 fungal ASVs associated with one or both metrics (Fig. 7). Classification with the FungalTraits database showed that five of these had a primary lifestyle as plant pathogens and two as litter saprotrophs, and the remaining two could not be assigned to a functional guild (Table S1). The Ascomycota were the most-abundant phylum with eight ASVs, with the remaining ASV belonging to the Basidiomycota. Orders represented by multiple ASVs included the Hypocreales (2), Pleosporales (2), and Glomerellales (2). One ASV belonging to the class Dothideomycetes had a far higher relative abundance across samples than the other ASVs (Fig. 7).

## DISCUSSION

A combination of factors was likely responsible for contrasting resistance to *P. palmivora* between two accessions of the cacao cultivar GNV-164 II, including phyllosphere bacterial and fungal diversity and the presence of specific beneficial and deleterious taxa. This variation in the MITC-331 and MITC-333 microbiomes may have been driven in part by light exposure and host nutrition, although the limitations of this pilot study prevent direct analysis of those environmental effects.

In support of the hypothesized differences in both individual microbial taxa and community-level properties of the microbiome, we observed higher prokaryotic but lower fungal alpha diversity in MITC-331 (Fig. S2), significant differences in phyllosphere community composition for fungi (Fig. 3 and 4), and six prokaryotic and 82 fungal ASVs whose relative abundance differed between cacao accessions contrasting in *Phytophthora* resistance (Fig. 5 and 6). Higher prokaryotic diversity was observed in the more resistant field accession MITC-331, a result that is consistent with a comparison of two cacao cultivars contrasting in resistance to the cacao pathogen *Moniliophthora perniciosa*. In that greenhouse study, the resistant cultivar CCN-51 had higher phylloplane bacterial diversity than the more susceptible cultivar Catongo (58). A diverse prokaryotic phylloplane community may be protective against pathogens independent of host genotype, perhaps by niche exclusion or the greater likelihood that a diverse community contains taxa capable of inhibiting pathogens. However, *lower* fungal diversity was observed in the phyllosphere of MITC-331 than MITC-333 (Fig. S2). The high prevalence of plant pathogens among the taxa correlated with lesion development serves as a reminder that biodiversity *per se* is not always beneficial to plant health if the taxa increasing diversity are pathogenic or facilitate pathogens.

Phyllosphere microbiomes isolated from MITC-331 and MITC-333 contrasted not only in diversity, but also in overall composition (Fig. 3 and 4) and relative abundance of individual taxa (Fig. 5 and 6), some of which may have played protective or disease-facilitative roles. Four of the six prokaryotic ASVs found at higher abundance in the MITC-331 phyllosphere belonged to the family Lachnospiraceae. The Lachnospiraceae were found to be one of three dominant prokaryotic families during anaerobic soil disinfestation (59), a management practice employed against soil-borne pathogens including other species of *Phytophthora*, e.g., in pepper (60). Despite the lack of quantitative correlation between the relative abundance of these ASVs and AUDPC or lesion size at day 5, their presence may have helped to inhibit *P. palmivora* in the phyllosphere of MITC-331 and this family is worth investigating as potential biocontrol agents. Identifying the origin of specific taxa in these phyllosphere microbiomes was beyond the scope of this study, but differences in relative abundance may have arisen from pod and seed microbiomes, environmental variables or host nutrition (as described later in this section), or stochastic effects.

Other taxa may have facilitated infection by *P. palmivora,* as suggested by positive correlations of their relative abundance with increased AUDPC and lesion size at day 5 and/or their classification as pathogens. Fifty percent of fungal ASVs preferentially associated with MITC-333 and 56% of the fungal ASVs whose abundance was positively correlated with lesion development were primarily classified as plant pathogens by the FungalTraits and FUNguild databases, with 96-100% of remaining classifiable fungi identified as saprotrophic (Table S1). The presence of multiple additional fungal pathogens may have made it easier for *P. palmivora* to infect MITC-333 leaves and cause lesions. Similar dynamics occur in maize, *Arabidopsis*, and alfalfa, where infection with some pathogens has been shown to suppress host defense systems, making them more susceptible to subsequent infections (61 – 64). This pathogen triggered susceptibility results from the downregulation of defense-related genes and suppression of secondary defense metabolite biosynthesis (62, 63).

Contrary to the hypothesis that we could identify potentially disease-protective taxa or microbiome features, only one of the eight microbial ASVs associated with disease progression was correlated with reduced lesion development (Fig. 7). This ASV could only be identified as belonging to the class Dothideomycetes, the largest class within the largest phylum of fungi (65), making it difficult to characterize any potentially protective traits. None of the numerous bacterial biocontrol agents successfully tested against *Phytophthora* spp. in cocoa [e.g., *Paenibacillus polymyxa* (19), *Bacillus amyloliquefaciens*, *Pseudomonas aeruginosa*, *Chryseobacterium proteolyticum* (66)] were found among the ASVs that differed in relative abundance between the two accessions or were correlated with lesion development. Of the fungi tested as anti-*Phytophthora* biocontrol agents in cacao [*Aspergillus* sp., *Penicillium* sp. (18), *Trichoderma* sp. (17), *Pestalotiopsis* sp., *Curvularia* sp., *Tolypocladium* sp., and *Fusarium* sp. (67)], only *Fusarium* sp. was present in the fungal ASVs contrasting in relative abundance between the two accessions, and all three ASVs were present in extremely low abundance (Fig. 7).

Environmental conditions including light exposure and host nutrition may have contributed to contrasting resistance between accessions, though replicates were too limited to assess their effects directly. MITC-331 received more light than MITC-333, potentially contributing to the differences in fungal communities. Incident light affects photoreceptors of both plants and microorganisms directly, as well as indirectly, modifying environmental properties on and around the leaf surface. However, the precise ways in which light impacts the establishment of the phyllosphere microbiome depend on the host plant species and microbial community of interest (42), and cacao-specific studies are lacking. In concordance with the finding here that more fungal than bacterial ASVs differed between field accessions, a greenhouse study of sunflower (*Helianthus annuus*) showed that fungal microbiomes were more responsive than bacterial microbiomes to light conditions (68). Many light-responsive elements that control processes such as virulence, conidia formation, or appearance of sclerotia have been identified at the molecular level in fungi (42). The link between light exposure, fungal phyllosphere communities, and *P. palmivora* incidence should be further investigated in a large-scale field study.

Host nutrition may also have played a role in the contrasting resistance between accessions. MITC-333 had higher leaf concentrations of potassium as well as multiple micronutrients, while MITC-331 had higher concentrations of calcium. Potassium is linked to disease resistance via its effects on jasmonic acid (JA) signaling. JA production increases in the absence of sufficient potassium, helping to provide resistance to JA-sensitive pathogens (69), a category that includes the *Phytophthora* spp. that cause BPR on cacao (70). Leaf concentrations of potassium below 1.00–1.20% are categorized as deficient in cacao (71), a range that includes MITC-331 (0.91% averaged across canopy positions) but not MITC-333 (1.35%). Lower concentrations in MITC-331 leaves of micronutrients including manganese and iron (Table 1) could also have played a role, as cacao trees infected by a fungal pathogen were found to be lower in these micronutrients than healthy trees in one field study .(72)

Though limited in scope to two field accessions of the genotype GNV-164 II, this study provides preliminary findings to guide future work. A similar study assessing light exposure, host nutrition, and phyllosphere microbiome characteristics in field-grown cacao, e.g., within clonal trials to control for the effect of genotype, would be beneficial in understanding whether the same factors control resistance to *P. palmivora* in the field. Larger host-microbiome datasets could also aid in identifying host-specific and generalist taxa that may contribute to biocontrol of cacao pathogens and understanding whether protective taxa are heritable or could be applied in the field. Both taxon-level and community-level features of the phyllosphere microbiome should be considered an important part of integrated pest management approaches to controlling *P. palmivora* on cacao.

## SUPPLEMENTAL MATERIAL

Supplemental figures and tables msphere.00013-23-s0001.docx Fig. S1, Fig. S2, Table S1.

ASM does not own the copyrights to Supplemental Material that may be linked to, or accessed through, an article. The authors have granted ASM a non-exclusive, world-wide license to publish the Supplemental Material files. Please contact the corresponding author directly for reuse.
