# Supplementary material for: Phyllosphere microbial diversity and specific taxa mediate within-cultivar resistance to Phytophthora palmivora in cacao
Source: mSphere. 2023 Aug 21;8(5):e00013-23. doi: 10.1128/msphere.00013-23 (PMC10597403; doi:10.1128/msphere.00013-23)
Supplement: Supplemental figures and tables — Fig. S1, Fig. S2, Table S1. [file msphere.00013-23-s0001.docx]

**Supplementary Figures and Tables**


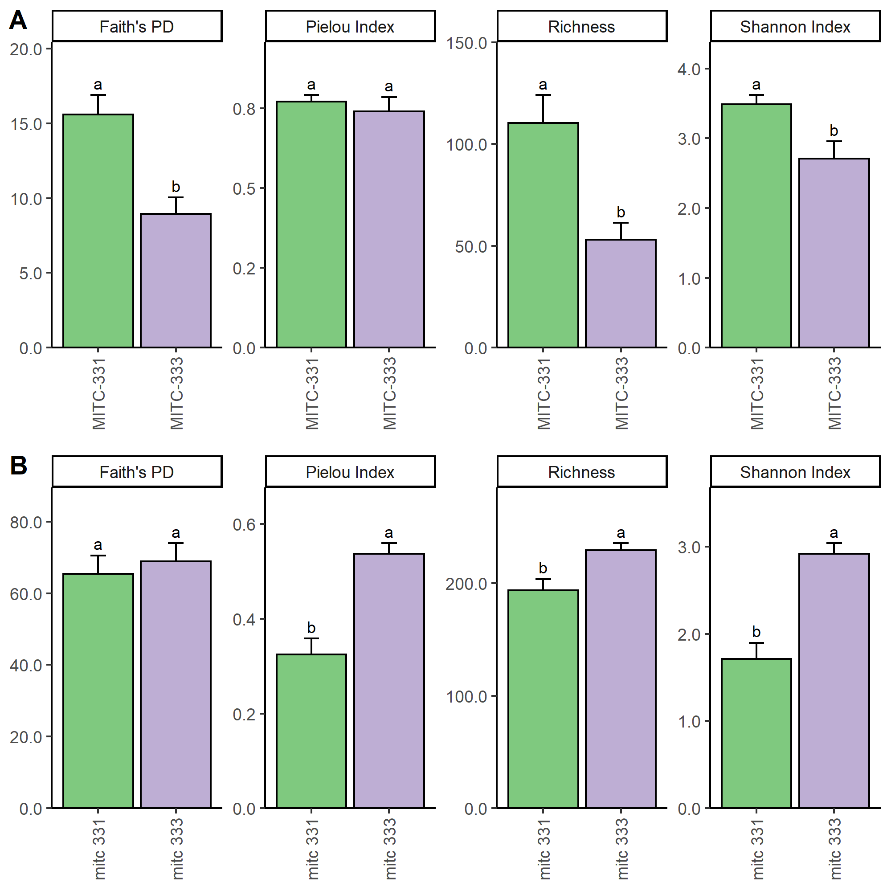


**Supplementary Fig. S1. Within-sample diversity of phyllosphere microbial communities.** A) Prokaryotic phyllosphere microbiome diversity differed between two accessions (MITC-331 and MITC-333) of the cultivar Gainesville II 164 for all metrics except the Pielou index. B) In contrast, fungal alpha diversity was higher in the phyllosphere of MITC-333 for all metrics except Faith’s phylogenetic distance. Error bars represent standard error.

**
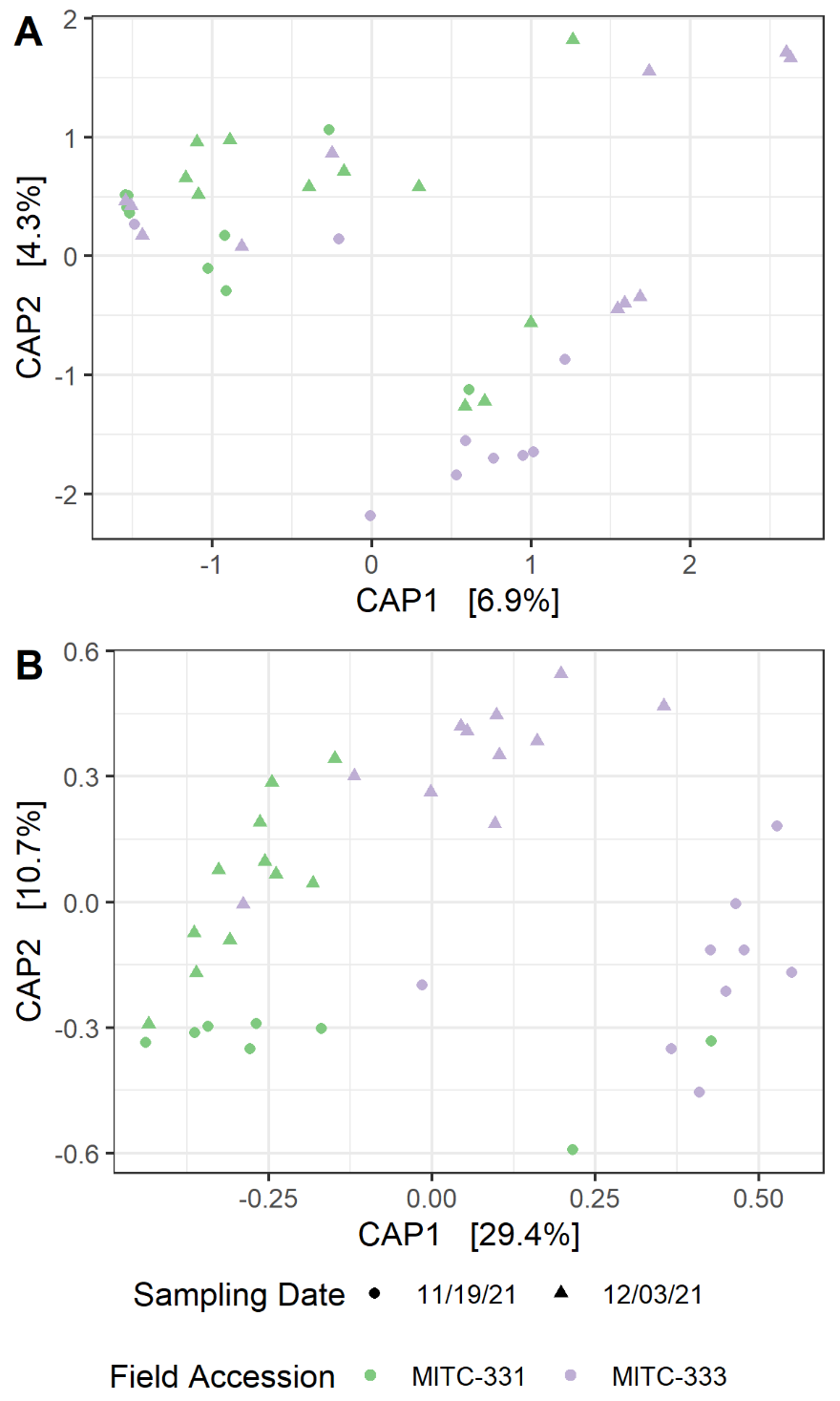
**

**Supplementary Fig. S2. Phylogeny-informed canonical analysis of principal coordinates (CAP) ordination**. A) Prokaryotic communities did not differ between field accessions MITC-331 and MITC-333 (p = 0.15) or by sampling date (p = 0.14). B) Fungal communities differed between accessions (p = 0.0002) and by sampling date (p = 0.0004). Ordinations were based on weighted UniFrac distance between relative abundance-transformed samples, using accession and sampling date as fixed factors.

**Supplementary Table 1: Functional guild classification of differentially abundant fungi.**

ASVs that differed in relative abundance between MITC-331 and MITC-333 were assigned to functional guilds using the FUNGuild and FungalTraits databases (Nguyen et al. 2016, Polme et al. 2021).

|  | FungalTraits | | | FUNGuild | |
| --- | --- | --- | --- | --- | --- |
|  | Primary Lifestyle | Secondary Lifestyle | Trophic Mode | Guild | Confidence Ranking |
| ASV102F-Ascomycota | NA | NA |  |  |  |
| ASV103F-Sordariomycetes | NA | NA |  |  |  |
| ASV109F-Fusarium | plant pathogen | litter saprotroph | Pathotroph-Saprotroph-Symbiotroph | Animal Pathogen-Endophyte-Lichen Parasite-Plant Pathogen-Soil Saprotroph-Wood Saprotroph | Possible |
| ASV10F-Gibberella | plant pathogen | litter saprotroph | Pathotroph | Plant Pathogen | Probable |
| ASV110F-Auricularia | wood saprotroph | | Saprotroph | Undefined Saprotroph | Probable |
| ASV111F-Cladosporium | litter saprotroph | plant pathogen | Pathotroph-Saprotroph-Symbiotroph | Animal Pathogen-Endophyte-Lichen Parasite-Plant Pathogen-Wood Saprotroph | Possible |
| ASV113F-Colletotrichum | plant pathogen | litter saprotroph | Pathotroph-Symbiotroph | Endophyte-Plant Pathogen | Probable |
| ASV117F-Vishniacozyma | soil saprotroph | | Pathotroph-Saprotroph-Symbiotroph | Fungal Parasite-Undefined Saprotroph | Possible |
| ASV119F-Tremellomycetes | NA | NA |  |  |  |
| ASV11F-Lectera | plant pathogen | | Pathotroph | Plant Pathogen | Probable |
| ASV128F-Neoconiothyrium | plant pathogen | litter saprotroph | |  |  |
| ASV12F-Colletotrichum | plant pathogen | litter saprotroph | Pathotroph-Symbiotroph | Endophyte-Plant Pathogen | Probable |
| ASV131F-Diaporthe | plant pathogen | litter saprotroph | Pathotroph-Symbiotroph | Endophyte-Plant Pathogen | Possible |
| ASV133F-Diaporthe | plant pathogen | litter saprotroph | Pathotroph-Symbiotroph | Endophyte-Plant Pathogen | Possible |
| ASV13F-Epicoccum | plant pathogen | litter saprotroph | Pathotroph-Saprotroph-Symbiotroph | Endophyte-Fungal Parasite-Lichen Parasite-Plant Pathogen-Wood Saprotroph | Probable |
| ASV149F-Plectosphaerellaceae | NA | NA |  |  |  |
| ASV14F-Nectriaceae | NA | NA | Pathotroph-Saprotroph-Symbiotroph | Animal Pathogen-Endophyte-Fungal Parasite-Lichen Parasite-Plant Pathogen-Wood Saprotroph | Possible |
| ASV151F-Epicoccum | plant pathogen | litter saprotroph | Pathotroph-Saprotroph-Symbiotroph | Endophyte-Fungal Parasite-Lichen Parasite-Plant Pathogen-Wood Saprotroph | Probable |
| ASV153F-Bipolaris | plant pathogen | litter saprotroph | Pathotroph | Plant Pathogen | Probable |
| ASV158F-Phaeosphaeriaceae | NA | NA | Pathotroph-Saprotroph | Fungal Parasite-Plant Pathogen-Plant Saprotroph | Probable |
| ASV15F-Allophoma | plant pathogen | | Pathotroph | Plant Pathogen | Probable |
| ASV165F-Fusarium | plant pathogen | litter saprotroph | Pathotroph-Saprotroph-Symbiotroph | Animal Pathogen-Endophyte-Lichen Parasite-Plant Pathogen-Soil Saprotroph-Wood Saprotroph | Possible |
| ASV16F-Arxiella | litter saprotroph | plant pathogen | Pathotroph-Saprotroph | Plant Pathogen-Undefined Saprotroph | Probable |
| ASV17F-Colletotrichum | plant pathogen | litter saprotroph | Pathotroph-Symbiotroph | Endophyte-Plant Pathogen | Probable |
| ASV18F-Lasiodiplodia | plant pathogen | foliar endophyte | Pathotroph | Plant Pathogen | Probable |
| ASV190F-Didymella | plant pathogen | litter saprotroph | Pathotroph-Saprotroph | Animal Pathogen-Plant Pathogen-Undefined Saprotroph | Probable |
| ASV197F-Dendryphiella | plant pathogen | | Pathotroph | Plant Pathogen | Probable |
| ASV1F-Dothideomycetes | NA | NA |  |  |  |
| ASV202F-Diaporthe | plant pathogen | litter saprotroph | Pathotroph-Symbiotroph | Endophyte-Plant Pathogen | Possible |
| ASV210F-Cladosporium | litter saprotroph | plant pathogen | Pathotroph-Saprotroph-Symbiotroph | Animal Pathogen-Endophyte-Lichen Parasite-Plant Pathogen-Wood Saprotroph | Possible |
| ASV219F-Pseudocoleophoma | plant pathogen | | Saprotroph | Undefined Saprotroph | Probable |
| ASV221F-Fusarium | plant pathogen | litter saprotroph | Pathotroph-Saprotroph-Symbiotroph | Animal Pathogen-Endophyte-Lichen Parasite-Plant Pathogen-Soil Saprotroph-Wood Saprotroph | Possible |
| ASV224F-Capnodiales | NA | NA |  |  |  |
| ASV238F-Diaporthe | plant pathogen | litter saprotroph | Pathotroph-Symbiotroph | Endophyte-Plant Pathogen | Possible |
| ASV23F-Diaporthe | plant pathogen | litter saprotroph | Pathotroph-Symbiotroph | Endophyte-Plant Pathogen | Possible |
| ASV249F-Dendryphiella | plant pathogen | | Pathotroph | Plant Pathogen | Probable |
| ASV24F-Teratosphaeriaceae | NA | NA | Pathotroph-Saprotroph | Animal Pathogen-Plant Pathogen-Undefined Saprotroph | Probable |
| ASV269F-Curvularia | plant pathogen | litter saprotroph | Pathotroph | Plant Pathogen | Probable |
| ASV26F-Colletotrichum | plant pathogen | litter saprotroph | Pathotroph-Symbiotroph | Endophyte-Plant Pathogen | Probable |
| ASV283F-Clonostachys | wood saprotroph | plant pathogen | Pathotroph | Plant Pathogen | Probable |
| ASV287F-Capnodiales | NA | NA |  |  |  |
| ASV28F-Diaporthe | plant pathogen | litter saprotroph | Pathotroph-Symbiotroph | Endophyte-Plant Pathogen | Possible |
| ASV300F-Orbiliaceae | NA | NA | Saprotroph | Wood Saprotroph | Probable |
| ASV302F-Articulospora | litter saprotroph | | Saprotroph | Undefined Saprotroph | Probable |
| ASV304F-Microdochium | plant pathogen | foliar endophyte | Pathotroph-Symbiotroph | Endophyte-Plant Pathogen | Possible |
| ASV33F-Coremiopassalora | litter saprotroph | | Pathotroph-Saprotroph | Plant Pathogen-Undefined Saprotroph | Probable |
| ASV340F-Buckleyzyma | epiphyte | litter saprotroph | Saprotroph | Undefined Saprotroph | Probable |
| ASV34F-Gibberella | plant pathogen | litter saprotroph | Pathotroph | Plant Pathogen | Probable |
| ASV35F-Diaporthe | plant pathogen | litter saprotroph | Pathotroph-Symbiotroph | Endophyte-Plant Pathogen | Possible |
| ASV39F-Memnoniella | litter saprotroph | | Saprotroph | Undefined Saprotroph | Possible |
| ASV3F-Vishniacozyma | soil saprotroph | | Pathotroph-Saprotroph-Symbiotroph | Fungal Parasite-Undefined Saprotroph | Possible |
| ASV40F-Rhodotorula | unspecified saprotroph | foliar endophyte | Pathotroph-Saprotroph | Animal Endosymbiont-Animal Pathogen-Endophyte-Plant Pathogen-Undefined Saprotroph | Probable |
| ASV42F-Roussoella | wood saprotroph | | Saprotroph | Undefined Saprotroph | Probable |
| ASV45F-Phaeosphaeriaceae | NA | NA | Pathotroph-Saprotroph | Fungal Parasite-Plant Pathogen-Plant Saprotroph | Probable |
| ASV4F-Colletotrichum | plant pathogen | litter saprotroph | Pathotroph-Symbiotroph | Endophyte-Plant Pathogen | Probable |
| ASV50F-Articulospora | litter saprotroph | | Saprotroph | Undefined Saprotroph | Probable |
| ASV51F-Eurotiomycetes | NA | NA |  |  |  |
| ASV52F-Cyphellophoraceae | NA | NA | Pathotroph-Saprotroph | Animal Pathogen-Undefined Saprotroph | Probable |
| ASV53F-Nectriaceae | NA | NA | Pathotroph-Saprotroph-Symbiotroph | Animal Pathogen-Endophyte-Fungal Parasite-Lichen Parasite-Plant Pathogen-Wood Saprotroph | Possible |
| ASV58F-Didymella | plant pathogen | litter saprotroph | Pathotroph-Saprotroph | Animal Pathogen-Plant Pathogen-Undefined Saprotroph | Probable |
| ASV59F-Uwebraunia | litter saprotroph | | Saprotroph | Undefined Saprotroph | Probable |
| ASV5F-Acremonium | unspecified saprotroph | foliar endophyte | Pathotroph-Saprotroph-Symbiotroph | Animal Pathogen-Endophyte-Fungal Parasite-Plant Pathogen-Wood Saprotroph | Possible |
| ASV60F-Diaporthe | plant pathogen | litter saprotroph | Pathotroph-Symbiotroph | Endophyte-Plant Pathogen | Possible |
| ASV61F-Neofusicoccum | plant pathogen | litter saprotroph | Pathotroph | Plant Pathogen | Probable |
| ASV63F-Setophaeosphaeria | plant pathogen | litter saprotroph | Pathotroph-Saprotroph | Fungal Parasite-Plant Pathogen-Plant Saprotroph | Probable |
| ASV666F-Basidiomycota | NA | NA |  |  |  |
| ASV68F-Colletotrichum | plant pathogen | litter saprotroph | Pathotroph-Symbiotroph | Endophyte-Plant Pathogen | Probable |
| ASV69F-Colletotrichum | plant pathogen | litter saprotroph | Pathotroph-Symbiotroph | Endophyte-Plant Pathogen | Probable |
| ASV6F-Gibberella | plant pathogen | litter saprotroph | Pathotroph | Plant Pathogen | Probable |
| ASV70F-Colletotrichum | plant pathogen | litter saprotroph | Pathotroph-Symbiotroph | Endophyte-Plant Pathogen | Probable |
| ASV71F-Cladosporium | litter saprotroph | plant pathogen | Pathotroph-Saprotroph-Symbiotroph | Animal Pathogen-Endophyte-Lichen Parasite-Plant Pathogen-Wood Saprotroph | Possible |
| ASV75F-Ascomycota | NA | NA |  |  |  |
| ASV76F-Plectosphaerella | plant pathogen | litter saprotroph | Pathotroph | Plant Pathogen | Probable |
| ASV78F-Phaeosphaeria | litter saprotroph | | Saprotroph | Undefined Saprotroph | Probable |
| ASV7F-Colletotrichum | plant pathogen | litter saprotroph | Pathotroph-Symbiotroph | Endophyte-Plant Pathogen | Probable |
| ASV86F-Acremonium | unspecified saprotroph | foliar endophyte | Pathotroph-Saprotroph-Symbiotroph | Animal Pathogen-Endophyte-Fungal Parasite-Plant Pathogen-Wood Saprotroph | Possible |
| ASV87F-Acrocalymma | wood saprotroph | | Saprotroph | Undefined Saprotroph | Probable |
| ASV88F-Diaporthe | plant pathogen | litter saprotroph | Pathotroph-Symbiotroph | Endophyte-Plant Pathogen | Possible |
| ASV89F-Cycasicola | litter saprotroph | wood saprotroph | Saprotroph | Wood Saprotroph | Probable |
| ASV8F-Nectriaceae | NA | NA | Pathotroph-Saprotroph-Symbiotroph | Animal Pathogen-Endophyte-Fungal Parasite-Lichen Parasite-Plant Pathogen-Wood Saprotroph | Possible |
| ASV99F-Cladosporium | litter saprotroph | plant pathogen | Pathotroph-Saprotroph-Symbiotroph | Animal Pathogen-Endophyte-Lichen Parasite-Plant Pathogen-Wood Saprotroph | Possible |
| ASV9F-Vishniacozyma | soil saprotroph | | Pathotroph-Saprotroph-Symbiotroph | Fungal Parasite-Undefined Saprotroph | Possible |
